# Canine Snake-Eye Myelopathy: Clinical, Magnetic Resonance Imaging, and Pathologic Findings in Four Cases

**DOI:** 10.3389/fvets.2019.00219

**Published:** 2019-07-05

**Authors:** John H. Rossmeisl, Thomas E. Cecere, Gregg D. Kortz, David A. Geiger, Richard L. Shinn, Jonathan Hinckley, David L. Caudell, Jessica A. Stahle

**Affiliations:** ^1^Veterinary and Comparative Neuro-Oncology Laboratory, Department of Small Animal Clinical Sciences, Virginia-Maryland College of Veterinary Medicine, Virginia Tech, Blacksburg, VA, United States; ^2^Department of Biomedical Sciences and Pathobiology, Virginia-Maryland College of Veterinary Medicine, Virginia Tech, Blacksburg, VA, United States; ^3^Department of Neurology, VCA Sacramento Veterinary Referral Center, Sacramento, CA, United States; ^4^Geiger Veterinary Neurology, Redwood City, CA, United States; ^5^Department of Pathology and Comparative Medicine, School of Medicine, Wake Forest University, Winston-Salem, NC, United States; ^6^Department of Diagnostic Imaging, Red Bank Veterinary Hospital, Tinton Falls, NJ, United States

**Keywords:** canine, central cord syndrome, cervical spinal cord, myelomalacia, intervertebral disc disease

## Abstract

Intramedullary signal change (ISC) is a non-specific finding that is frequently observed on magnetic resonance imaging (MRI) examinations of the canine spinal cord. ISC can represent a variety of primary pathological processes such as neoplasms or myelitides or secondary changes such as edema, cysts, gliosis, or myelomalacia. An unusual phenotype of ISC is the “snake-eye” myelopathy (SEM), which refers to bilaterally symmetric T2 hyperintensities preferentially affecting the ventral horn gray matter on transverse MR images, which resemble a pair of snake's eyes. The pathophysiology of SEM is poorly understood in humans, and this imaging finding may be associated with cervical spondylotic myelopathy, spinal cord ischemia, ossification of the posterior longitudinal ligament, amyotrophic lateral sclerosis, and Hirayama disease. Here we describe four dogs with cervical MRI examinations consistent with an SEM-like phenotype. All dogs initially presented with a central cord syndrome or tetraparesis referable to a C6-T2 neuroanatomic localization, which was attributed to disc-associated spinal cord compression in three cases, while one dog had the SEM-like phenotype with no identifiable etiology. Once the SEM-like phenotype was present on MRI examinations, dogs demonstrated insidious clinical deterioration despite therapeutic interventions. Deterioration was characterized by lower motor neuron weakness and neurogenic muscle atrophy progressing to paralysis in the thoracic limbs, while neurological functions caudal to the level of the SEM-like lesion remained largely preserved for months to years thereafter. Neuropathological features of the SEM-like phenotype include multisegmental cavitations and poliomyelomalacia of laminae VI-IX of the caudal cervical spinal cord, although the lesion evolved into pan-necrosis of gray matter with extension into the adjacent white matter in one case with an 8 years history of progressive disease. Although the pathophysiology of SEM remains unknown, the topographical distribution and appearance of lesions is suggestive of a vascular disorder. As the SEM-like phenotype was uniformly characterized by longitudinally and circumferentially extensive neuronal necrosis, results of this small case series indicate that dogs with clinical signs of central cord syndrome and the SEM-like phenotype involving the cervicothoracic intumescence on MR examinations have a poor prognosis for the preservation or recovery of thoracic limb motor function.

## Introduction

Intramedullary signal changes (ISC) are often observed during magnetic resonance imaging (MRI) examinations of humans and dogs with a variety of spinal cord diseases (1–7). To refine neuroradiological diagnoses and provide prognostic information, attempts are made to differentiate ISC into cystic/cavitary (CISC) and non-cavitary (NCISC) types based on the appearance and topographical distribution of lesions along the vertebral column and within the spinal cord parenchyma (3, 5–7). CISC, common types of which are post-traumatic cystic change and syringomyelia (SM), are typically described as well delineated or sharply marginated tubular or loculated intraparenchymal lesions that are T1W hypointense and whose T2W and short tau inversion recovery (STIR) signals are iso- to slightly hypointense relative to cerebrospinal fluid (CSF) (5, 7, 8). While it can be difficult to discriminate types of CISC purely using imaging criteria, it is widely accepted that focal cystic change occurs in proximity to the lesion epicenter, while SM extends into adjacent spinal segments and typically involves the central portion of the spinal cord parenchyma with extension into the dorsal horns (8). NCISC can occur in any cross-sectional topographic distribution in the spinal cord, generally appear as poorly marginated or ill-defined intramedullary lesions whose T2W signal is relatively hypointense compared to CSF, and can be T1W isointense or hypointense relative to normal neural parenchyma depending on the cause and duration of disease. There have been relatively few reports correlating the imaging appearances of CISC or NCISC to neuropathological findings in dogs with naturally occurring neurological disease, and the majority of these have occurred in the context of SM secondary to Chiari-like malformation (CM) or spinal cord injury (SCI) associated with intervertebral disc herniation (IVDH) or protrusion (3, 8). Based on these and other human studies, a range of pathological processes can result in ISC including atrophy, cystic or cavitary necrosis, edema, demyelination, gliosis, hemorrhage, and SM (3, 8, 9).

**Figure 1:**
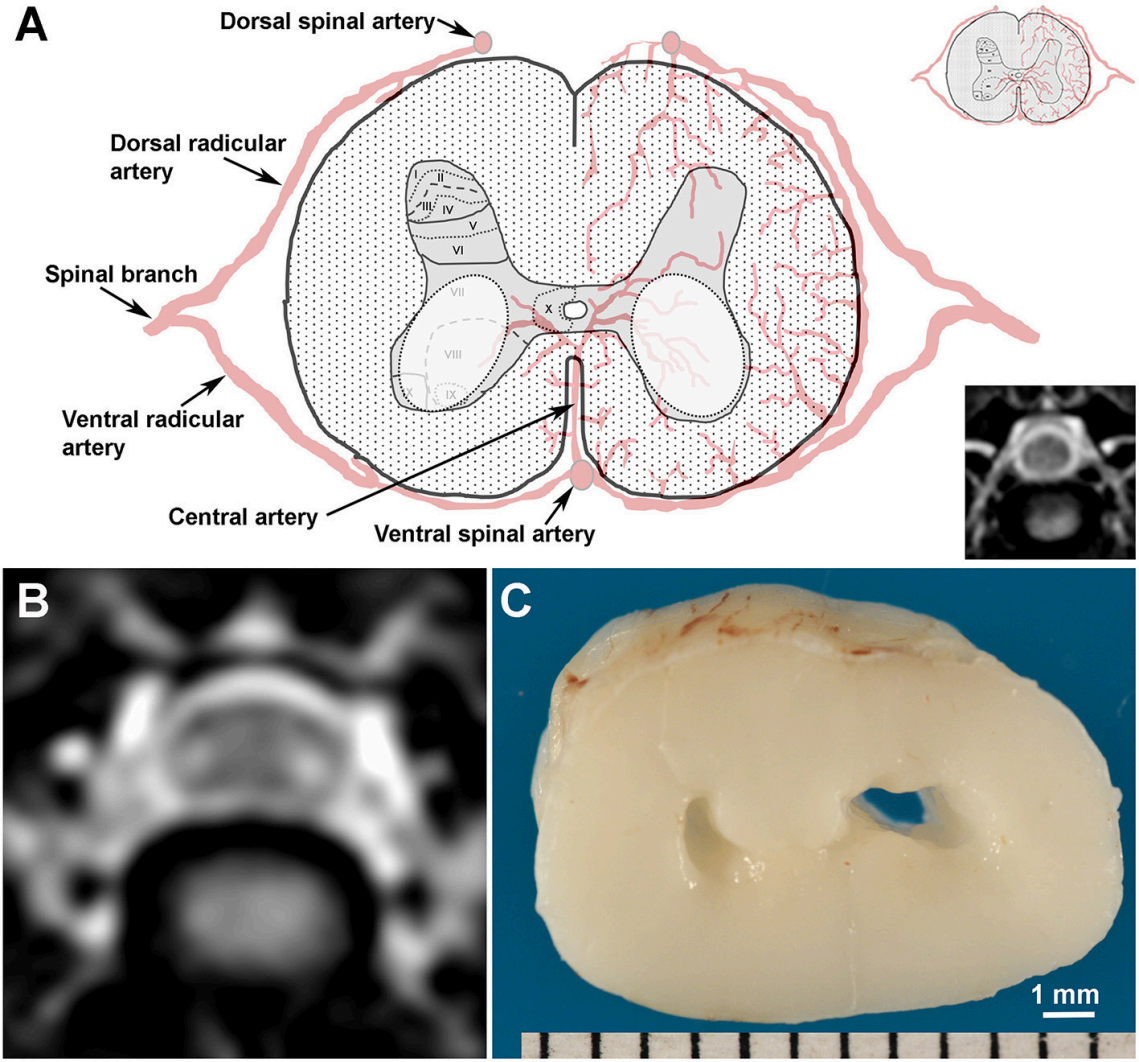
Topography, MRI, and gross pathological appearance of canine snake eye myelopathy (SEM)-like phenotype. **(A)** Schematic and normal T2W transverse MR image (lower inset) of canine C5 spinal cord segment illustrating putative arterial supply and Rexed laminar structure of the gray matter (upper inset). SEM-like lesions appear as bilaterally symmetric lesions (white dashed ovals) distributed in laminae VI-IX of the gray matter. **(B)** Transverse T2W MRI through the cranial aspect of C5 from Case 1 with the SEM-like phenotype demonstrating bilaterally symmetric intramedullary hyperintensities in the ventral horn gray matter and dorsoventral flattening of the spinal cord. **(C)** The SEM-like phenotype is pathologically characterized by bilateral cavitations in the C5 ventral horns and poliomalacia; Case 1.

Despite limited data regarding pathological correlates of ISC, qualitative and quantitative evaluations of ISC are emerging as prognostic indicators in dogs with spinal cord disease. In dogs with acute SCI secondary to IVDH, the presence and degree of T2W-ISC have been reported as negative prognostic indicators (2, 10, 11). In addition, T2W-ISC has been associated with the severity and chronicity of clinical signs in dogs with cervical spondylomyelopathy (CSM) (4), and chronic changes such as gliosis or fibrosis that occur during evolution or progression of ISC have been correlated with the development or intensification of clinical signs in dogs with CM-SM (8). In humans, one uncommon manifestation of ISC is “snake-eye” myelopathy (SEM), which has also been referred to as “owls-eye” myelopathy. SEM is characterized by bilaterally symmetric T2 hyperintensities preferentially affecting the ventral horn gray matter on transverse MR images, which resemble a pair of snake's eyes (Figure 1) (9). In SEM, lesions may be iso- or hypointense on T1W images, and typically do not display contrast enhancement. However, imaging manifestations of SEM are non-specific, as this entity has been observed in cases of cervical spondylotic myelopathy, sporadic lower motor neuron disease, spinal cord ischemia, ossification of the posterior longitudinal ligament, amyotrophic lateral sclerosis, cobalt toxicity, and Hirayama disease (9, 12). Humans with SEM manifest with symptoms of lower motor neuron arm weakness, with the prognosis related to the underlying etiology.

In this case series we describe four dogs with naturally occurring myelopathies with MRI examinations consistent with SEM, and annotate this novel imaging presentation in the cervical spinal cord with clinical signs, outcomes, and neuropathological correlations.

## Prevalence of SEM

Medical records and cervical vertebral column MRI examinations from 481 dogs with C6-T2 neuroanatomic diagnoses were reviewed for the purposes of this study (see Supplementary Materials and Methods). Etiologic diagnoses identified on these MRI imaging studies included IVDH (339/481; 70%), CSM (32/481; 7%), fibrocartilagenous embolic myelopathy (30/481; 6%), neoplasia (30/481; 6%), meningomyelitis or myelitis (21/481; 4%), discospondylitis (18/481; 3.5%), undetermined (7/481; 1.5%), and vertebral fracture/subluxation (4/481; 0.08%). ISC was present in a total of 169/481 (35%) of these cervical MRI examinations, and ISC with an SEM-like phenotype was present in four dogs, yielding an SEM prevalence of 0.08% in dogs with a neuroanatomic diagnosis of C6-T2 myelopathy. All dogs in which an SEM-like phenotype was identified were imaged using 1.5T MRI systems (Supplementary Data Sheet 1).

## Case Presentations and Outcomes

### Case 1

A 6 years old, neutered male Lhasa Apso was presented with a 1 month history of progressive gait dysfunction characterized by the owner as an “army crawling” appearance. The dog's initial clinical presentation included a low head carriage, ataxia of all limbs, and suspected cervical hyperpathia. The cervical hyperpathia and ataxia resolved with a 2 weeks course of treatment with carprofen and cage rest, but over the subsequent weeks the gait evolved into what the owner described as “army crawl” (Supplementary Video 1).

Significant physical examination abnormalities were limited to the nervous system. The dog was ambulatory but tetraparetic, with weakness in the thoracic limbs more severe than in the pelvic limbs. Thoracic limb weight bearing occurred on the antebrachii. Thoracic limb postural reaction deficits and paresis were present and associated with reduced thoracic limb muscle tone, flexor withdrawal, and triceps reflexes bilaterally. Pelvic limb proprioception and postural reactions were delayed in the left pelvic limb and normal in the right pelvic limb. The pelvic limb spinal reflexes were intact. No apparent cervical hyperpathia was observed. Moderate muscle atrophy was present in the thoracic limbs. Neuroanatomic diagnoses included C6-T2 myelopathy with central cord component or bilateral brachial plexus neuropathy.

The dog was anesthetized and electromyographic (EMG) examination of the left cervical region and forelimb and an MRI of the cervical vertebral column were performed. Increased insertional activity, fibrillation potentials, and positive sharp waves were identified on EMG of the left triceps, biceps brachii, extensor carpi radialis, and supraspinatus muscles. No abnormal EMG findings were noted in muscles of the head, thoracolumbar epaxial region, or left pelvic limb. The MRI revealed extradural T2W hypointense material in the ventral vertebral canal overlying the C6-C7 disc space consistent with IVDH resulting in moderate ventral and left-sided extradural compression and dorsal displacement of the spinal cord. On sagittal T2W and STIR images, a well-defined, linear intramedullary hyperintensity was identified that extended from C5 to the cranial aspect of C7. On transverse T2W (Figure 1B) and FLAIR images, the hyperintensities were bilaterally symmetric and restricted to the ventral gray matter. These hyperintensities were isointense on T1W images and non-enhancing following IV gadolinium administration. The neuroradiological diagnosis was C6-C7 intervertebral disc extrusion with associated mutisegmental ISC. The distribution of the ISC was interpreted as compatible with a bilateral ventral horn lesion, with an SEM-like phenotype as described in humans (9). Differential diagnoses for the observed ISC included cavitations, edema, gliosis, and necrosis.

Ventral slot decompression was performed at C6-C7. The dog was discharged to the care of the owner 2 days after surgery with no change in its neurological status. An examination performed 2 weeks later revealed worsening thoracic limb paresis and muscle atrophy. A computed tomographic scan of the cervical spine revealed no residual extradural compression at C6-C7 or other complications associated with the ventral slot procedure. A physical therapy program was initiated. Despite rehabilitation, thoracic limb motor function gradually declined over the subsequent 4 weeks at which point the dog was euthanized when non-ambulatory from thoracic limb paralysis. A necropsy was performed.

On gross examination, there was no evidence of extruded intervertebral disc material in the vertebral canal, and a ventral slot defect filled with fibrous tissue was present at C6-C7. Bilateral cavitary lesions (Figure 1C) involving ventral horn gray matter was observed extending from the cranial aspect of the C5 through the C8 spinal cord segments. On microscopic examination, the lesions were somewhat symmetric in appearance and restricted to laminae VI-IX of the gray matter, but were most severe within the C5-C7 spinal cord segments. Cavitary regions in gray matter, which were most conspicuous in lamina VI, the central aspect of lamina VII and the dorsomedial aspect of lamina VIII, contained cellular processes, gitter cells, astrogliosis, faintly eosinophilic and shrunken ghost neurons lacking nuclear staining, and rare chromatolytic neurons. Neurons in the lateral portions of laminae VII, central portion of lamina VIII, and in laminae IX displayed acidophilic neuronal necrosis characterized by shrunken, angular and hypereosinophilic cytoplasm, and nuclear pyknosis. No fibrocartilaginous emboli were detected with Alcian blue staining. The final diagnosis was regionally extensive, bilaterally symmetric, chronic gray matter necrosis in spinal cord segments C5-C8 resulting in neurogenic thoracic limb muscular atrophy and fibrosis.

### Case 2

An 8 years old, neutered male mixed breed dog was evaluated for a 3 days history of cervical hyperpathia and right hemiparesis that progressed to tetraplegia. Upon presentation, the dog was tetraplegic with absent postural reactions in all limbs, diminished muscle tone and hyporeflexia in the thoracic limbs, and pelvic limb spasticity and hyperreflexia. Cervical hyperpathia was apparent on flexion of the neck. The neuroanatomic diagnosis was C6-T2 myelopathy.

**Figure 2:**
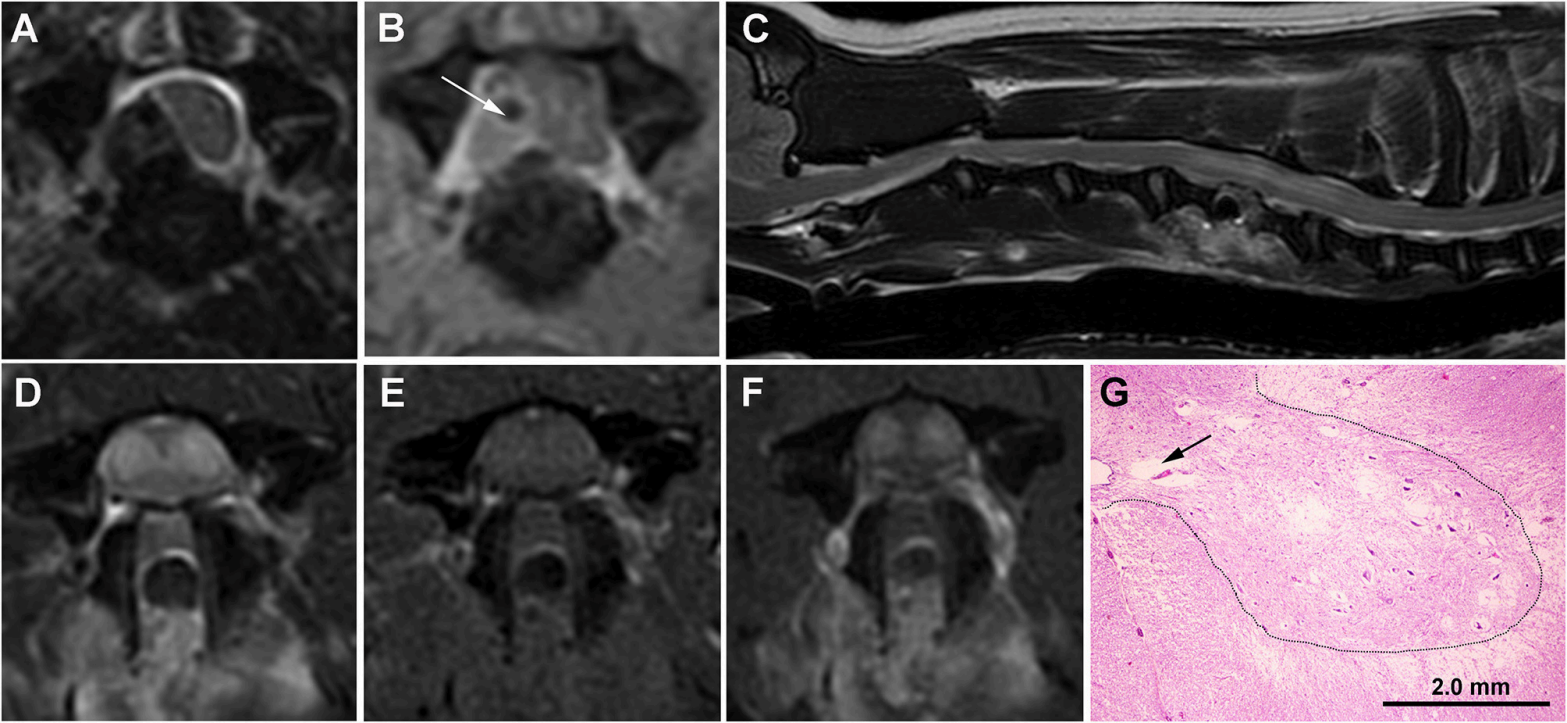
Serial MRI and neuropathology of Case 2. **(A)** Transverse T2W image with hypointense extradural lesion at C5-C6 disc space, lateralized to the right aspect of the vertebral canal, and causing marked compression and displacement of the spinal cord. The lesion is of mixed signal intensity on transverse TIW post-contrast images **(B)** suggesting the presence of extradural hemorrhage admixed with hypointense extruded disc (white arrow). **(C)** Sagittal T2W image obtained 7 days after surgery demonstrating the C5-C6 ventral slot defect and a tubular hyperintense intramedullary lesion extending from the cranial aspect of C5 to the cranial aspect of C7. On transverse images, the SEM-like phenotype appears as bilaterally symmetric T2W hyperintense **(D)** gray matter lesions that are mildly T1W hyperintense **(E)** and contrast-enhancing **(F)**. Despite resolution of the previous extradural compression, the spinal cord throughout the lesion region is swollen with attenuation of the subarachnoid space. **(G)** Histology of the C6 spinal cord demonstrating various stages of cavitations (black arrow) and gray matter necrosis, with neurons in the lateral portions of laminae VII and IX displaying acidophilic necrosis characterized by angular and hypereosinophilic cytoplasm and nuclear pyknosis. At the junction of the intermediate and ventral gray horns (dashed line) with spinal cord white matter, there is also white matter vacuolization. H&E stain.

An MRI of the cervical spine was performed under general anesthesia, the results of which were consistent with C5-C6 IVDH with associated compressive extradural hemorrhage (Figures 2A,B). No abnormal changes were observed within the spinal cord parenchyma. Ventral slot decompression was performed at C5-C6. The dog was ambulatory with pelvic limb ataxia when it was discharged to the owner 3 days after surgery.

Seven days after surgery, the dog represented non-ambulatory with severe lower motor neuron paresis and muscle atrophy in the thoracic limbs, with the pelvic limbs being neurologically intact. A cervical MRI examination was repeated and complete resolution of the previously identified extradural compression at C5-C6 was noted. However, contrast-enhancing ISC with an SEM-like phenotype was observed (Figures 2C–F), and subacute reperfusion injury, intramedullary hemorrhage, edema, myelitis, or infarction considered possible etiologies for the imaging abnormalities. Lumbar CSF analysis revealed albuminocytologic dissociation (total protein 101 mg/dl; reference range <45 mg/dl). CBC, indirect blood pressure, buccal mucosal bleeding time, and coagulation profile were within reference ranges. The dog underwent physical therapy and treatment with prednisone (0.5 mg/kg/day PO for 10 days) but remained non-ambulatory. Thoracic limb motor function and muscle mass declined insidiously and the dog was euthanized and a necropsy performed 3 months after surgery. Pathological findings in the gray matter were similar to those described for Case 1, except that at the junction of the ventral and intermediate gray horns with white matter, vacuolization of the white matter associated with variable axonal swelling was observed (Figure 2G). The final diagnosis was multisegmental, bilaterally symmetric, chronic gray matter necrosis, spinal cord segments C5-C8.

### Case 3

**Figure 3:**
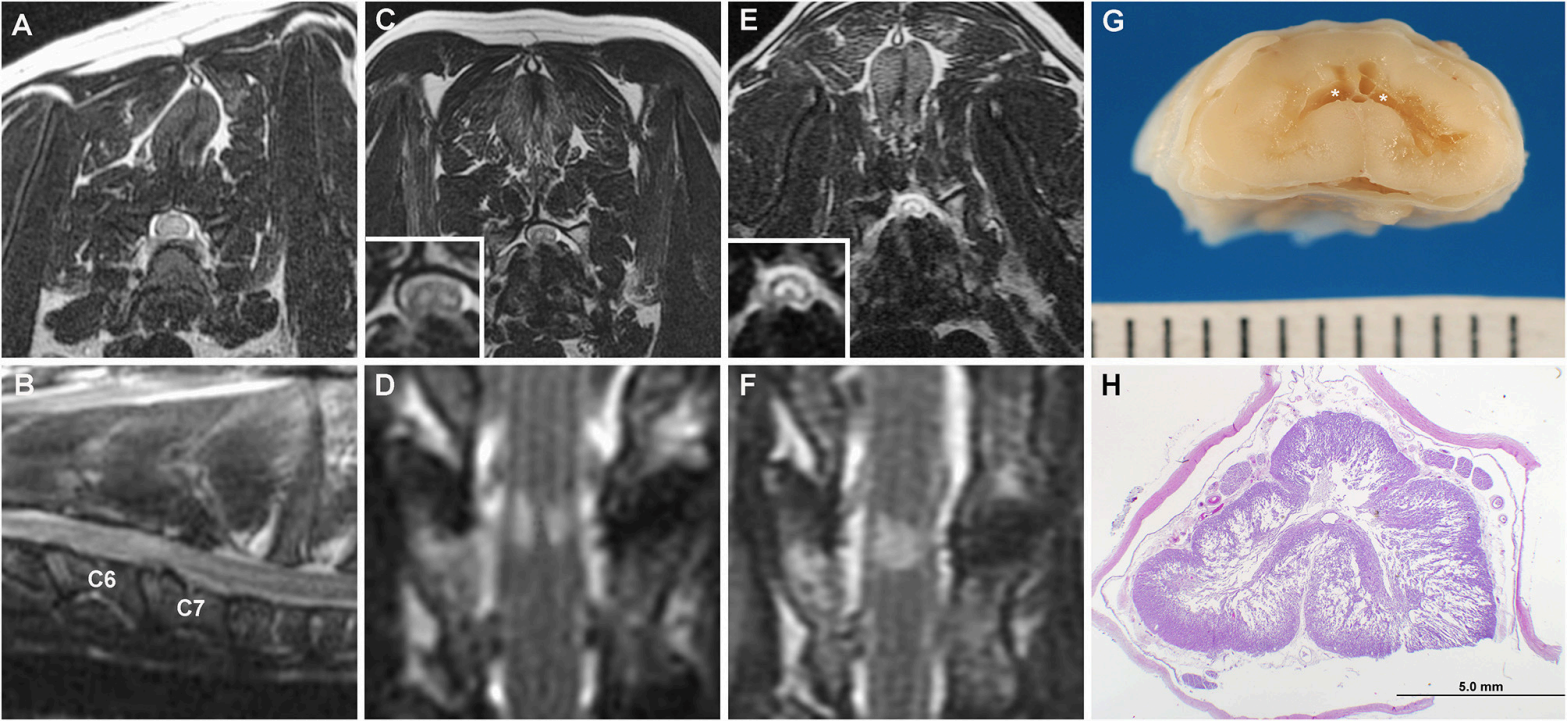
Serial MRI and neuropathology of Case 3. T2W transverse **(A)** and parasagittal images **(B)** at the time of initial diagnosis of C6-C7 CSM demonstrating no ISC within the spinal cord. Transverse **(C)** T2W and dorsal STIR **(D)** obtained 6 years after initial surgery revealing bilaterally symmetric hyperintense SEM-like lesions in the ventral gray matter overlying the C6-C7 disc space. On MRI examination obtained 2 years after panels **(C,D)**, the previously noted SEM-like lesions have coalesced into a singular T2W/STIR hyperintense intramedullary lesion involving all gray matter regions **(E,F)**, and spinal cord atrophy has also progressed at the C6-C7 site. **(G)** Geographic cavitation (asterisks) and gray matter necrosis is present in the gross specimen of the C6 spinal cord segment. **(H)** Bilateral cavitation and necrosis of the gray matter is evident on a histologic section of the C6 spinal cord with vacuolization extending into the surrounding white matter. H&E stain.

A 4 years old spayed female Doberman Pinscher was initially evaluated for ambulatory tetraparesis and cervical hyperpathia that localized to the C6-T2 spinal cord segments. A cervical MRI examination performed at that time revealed disc-associated CSM at C6-C7, with no abnormal ISC detected (Figures 3A,B). A ventral slot decompression was performed at C6-C7 and a complete neurological recovery was made.

The dog represented approximately 6 years later with recurrent ambulatory tetraparesis referable to a C6-T2 myelopathy and generalized muscle atrophy. A cervical MRI examination was repeated which revealed decreased *in situ* signal intensity involving all discs in the cervical region. There were mild disc protrusions at C5-C6 and C6-C7 resulting in minimal compression of the spinal cord. T2W hypointense material was noted dorsal to the spinal cord at C5-C6 and C6-C7 with minimal attenuation of the dorsal epidural fat and CSF signal. Bilaterally symmetric T2W/STIR hyperintensity consistent with an SEM-like phenotype was observed over the disc space of C6-C7 (Figures 3C,D), as were changes within the vertebral bodies of C6-C7 consistent with a previous ventral slot procedure. The MRI findings were interpreted as consistent with progression of CSM with adjacent segment disease and ligamentous hypertrophy. Muscle biopsies were performed to identify possible concurrent neuromuscular disorders that may have been causing or contributing to the observed generalized muscle atrophy. Muscle biopsies obtained from the triceps and biceps femoris revealed moderate generalized muscle atrophy and excessive intramyofiber lipid droplets in type 1 fibers, consistent with a metabolic myopathy secondary to oxidative disorder, carnitine deficiency, or endocrinopathy. The dog was diagnosed with hypothyroidism, and therapy with levothyroxine (0.1 mg/4.5kg PO q 12 h), acetyl-L-carnitine (50 mg/kg PO q 12 h), coenzyme Q10 (1 mg/kg PO q 24 h), riboflavin (5 mg/kg PO q 24 h), vitamin E (200 IU PO q 24 h), and gabapentin (5 mg/kg PO q 12 h) was initiated.

The dog was re-evaluated 10 months later for slowly progressive and severe thoracic limb weakness and muscle atrophy. The dog was weakly ambulatory with a neurological examination that remained consistent with C6-T2 myelopathy, although a central cord component or progression of the previously diagnosed generalized neuromuscular disorder were also suspected due to the preferential severity of weakness of the thoracic limbs. Euthyroidism was documented. A third cervical MRI was obtained, with findings similar to the previous examination except that SEM signal was present at C5-C6 and C6-C7, and spinal cord atrophy had progressed at both sites based on objective and serial measurements of spinal cord diameter at the lesion epicenters (3). Given the SEM-like findings suggestive of gliosis or poliomyelomalacia, additional surgical therapy was not pursued. Clinical signs continued to deteriorate and the dog became non-ambulatory with severe muscle atrophy 16 months later. A fourth cervical MRI examination documented additional progression of the CSM characterized by worsening disc protrusion and progressive spinal cord atrophy at C5-C6 and C6-C7. The previously distinct, bilaterally symmetric SEM signals at C5-C6 and C6-C7 had coalesced into singular T2W/STIR hyperintense lesions affecting the gray matter (Figures 3E,F). The dog was euthanized and a necropsy performed.

On gross examination, there was protrusion of the intervertebral discs into the vertebral canal at C4-C5, C5-C6, and C6-C7. There was a region of dorsoventral collapse and softening of the C6 cord segment. On sectioning, the C6 segment contained extensive bilateral cavitations, poliomyelomalacia, and tan discoloration of the gray matter (Figure 3G). The C5 and C7 segments were similarly affected, but not as severe.

Microscopic examination revealed that the cavitary processes extending from the C5-C7 segments largely obliterated the gray matter bilaterally and extended in a symmetric cribriform fashion far into adjacent white matter, with changes most extensive in the C6 segment (Figure 3H). The cavitary regions in gray matter consisted predominantly of unstained regions containing thin cellular processes, ill-defined granular debris, ghost form neurons, gitter cells, and astrogliosis. The vacuolated white matter contained some swollen axons, astrogliosis and loss of myelin in severely affected regions. The ventral spinal nerve roots in affected segments demonstrated marked endoneurial edema and fibrosis. No annulus fibrosis was observed between the C6-C7 vertebral bodies and the intervertebral disc space was occupied by a cartilaginous mass with basophilic matrix and relatively acellular connective tissue interpreted as mature fibrous connective tissue. The cartilaginous endplates were focally disrupted, with extension of fibrous tissue and cartilage within adjacent vertebral bodies. These fibrous and cartilaginous elements focally protruded into the vertebral canal, covered by a densely ossified dorsal longitudinal ligament. The final diagnosis was extensive bilateral cavitary pan-necrosis of gray matter, extending into white matter, spinal cord segments C5-C7, suspected secondary to CSM.

### Case 4

A 3 years old, neutered male, Boxer was evaluated for a 6 weeks history of progressive gait abnormality characterized by thoracic limb weakness. The owner reported that the dog's gait abnormalities developed in the absence of any known precipitating event. Neurological examination findings were similar to those described for Case 1, except the pelvic limb posture and gait were normal, such that the dog stood and ambulated in a prayer-type position. Additionally, the thoracic limb paresis, neurological deficits, and muscle atrophy were asymmetric with the right side being more severely affected than the left. The neuroanatomic diagnosis was C6-T2 myelopathy with central cord component or bilateral brachial plexus neuropathy.

**Figure 4:**
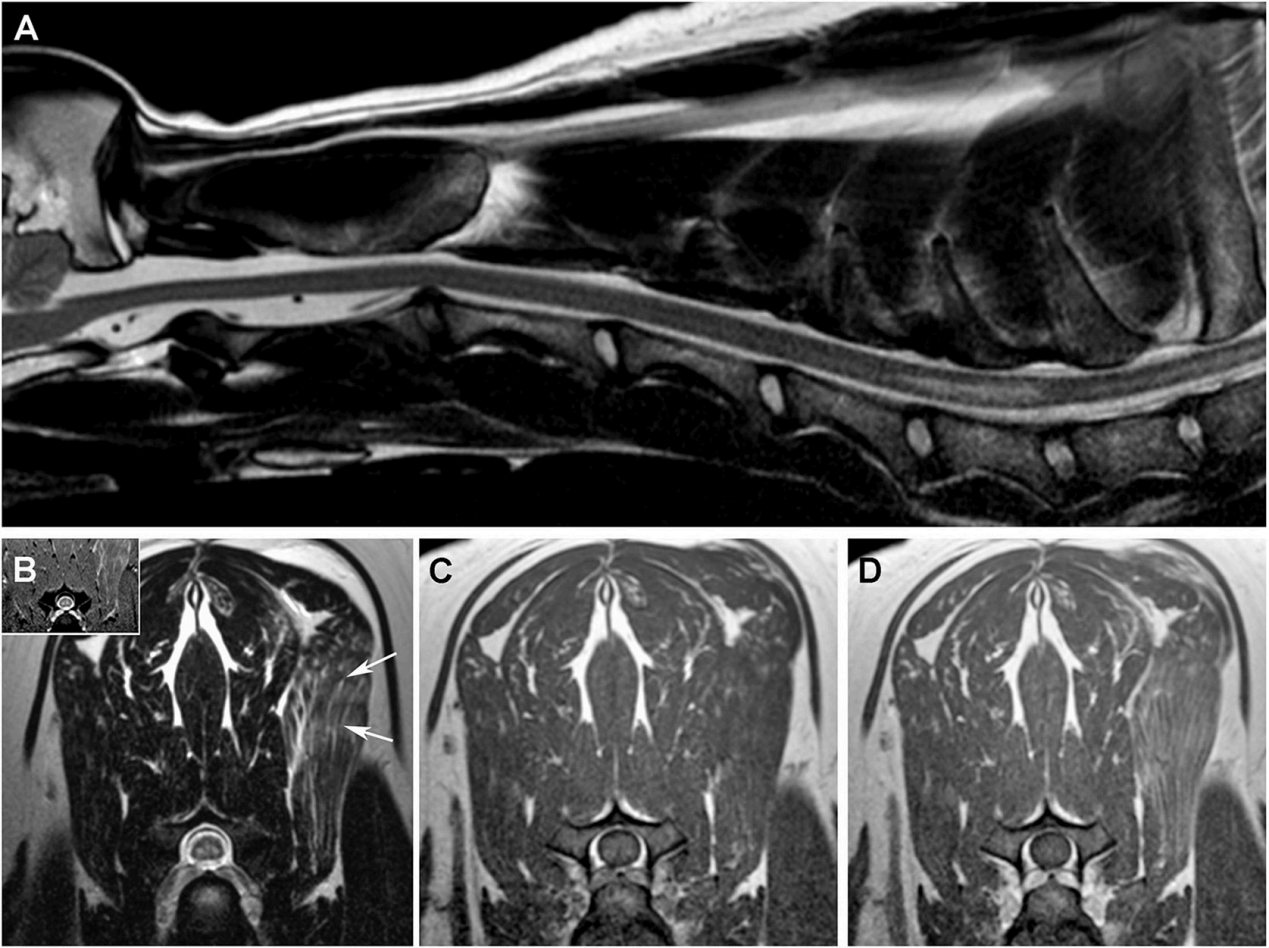
MRI features of the SEM-like phenotype, Case 4. **(A)** Sagittal T2W image with linear hyperintense intramedullary lesion extending from the cranial aspect of C6 to the cranial aspect of C7. On transverse images, SEM appears as bilaterally symmetric T2W and T2*GRE **(B)** hyperintense lesions in the ventral gray matter that are T1W isointense **(C)** and non-enhancing following intravenous gadolinium administration **(D)**. There is heterogeneously increased T2W signal [**(B)**, arrow] and contrast enhancement **(D)** of the left serratus ventralis muscle.

An MRI examination of the cervical spine was obtained under general anesthesia (Figure 4), with ISC with an SEM-like phenotype identified in the caudal cervical spinal cord. Cerebrospinal fluid was obtained via lumbar puncture, and albuminocytologic dissociation was the only observed CSF abnormality (total protein 68 mg/dl; reference range <45 mg/dl). Positive sharp waves were detected on EMG of the left triceps, rhomboideus, infraspinatus, and supraspinatus muscles. Infectious disease testing was performed including evaluation of serum antibody titers against toxoplasmosis, neosporosis, Ehrlichia canis, and rocky mountain spotted fever (Infectious Disease Laboratory, Athens, GA, USA). Serum was also tested for cryptococcal antigen (latex agglutination) and CSF for canine distemper virus (RT-PCR; Infectious Disease Laboratory). No infectious etiology was identified, and the dog was treated for presumptive immune-mediated myelitis with cyclosporine (5 mg/kg/day PO) and prednisone (1 mg/kg PO q 12 h), with no change in clinical status observed at the 1 week recheck examination.

Five weeks after diagnosis, the dog died shortly after being admitted to the hospital in septic shock secondary to elbow decubital ulcers. The owner reported that the dog's clinical condition had worsened since the last recheck, and thoracic limb muscle atrophy was notably more severe at this admission. A necropsy examination revealed a bilaterally symmetric chronic poliomyelomalacia involving the C5-C8 spinal cord segments and neurogenic muscular atrophy of the thoracic limb musculature, with gross and microscopic features closely recapitulating those reported for Case 1. No other lesions or etiology for the SEM-like phenotype were identified.

## Discussion

In this series, the SEM-like phenotype was a rare imaging manifestation of ISC that was clinically characterized by a central cord syndrome associated with progressive thoracic limb weakness and muscle atrophy. On MRI, SEM-like features included bilaterally symmetric intramedullary T2W hyperintensities in the caudal cervical spinal cord ventral horn gray matter, which correlated to regions of gray matter cavitations and necrosis affecting laminae VI-IX on neuropathologic examinations. This case series demonstrates that the clinicopathological features of the canine SEM-like phenotype overlap considerably with previous published descriptions of this entity in humans and dogs with experimentally induced cervical spinal cord compression (9, 12, 13).

The MRI appearance of the SEM-like phenotype of the three cases in this series with chronic clinical signs was similar to those classically described, with bilaterally symmetric ventral gray matter lesions demonstrating T2W/STIR hyperintensity, T1W isointensity, and no enhancement following gadolinium administration (9, 12, 13). Case 2 differed from other cases in that SEM-like lesions were identified 7 days after decompressive surgery for IVDH; noted in regions of spinal cord swelling rather than being associated with spinal cord compression or atrophy; involved larger regions of gray matter; and demonstrated contrast enhancement. In dogs and humans, subacute ischemic myelopathies can be associated with spinal cord swelling and contrast-enhancing ISC, and in humans subacute SEM may also demonstrate patchy enhancement (14, 15). Suspected reperfusion injury manifesting as SEM has been reported in humans following uncomplicated cervical surgery, which is similar to Case 2 in which the SEM-like phenotype developed despite apparently successful surgical intervention (16). In addition, upon necropsy examination of Case 2, lesions were restricted to the intermediate and ventral gray matter regions, indicating that the increased signal present in the dorsal horns of Case 2 on MRI represented a reversible pathological phenomenon, such as edema.

The topographical distribution and neuropathological features of ischemic change that were predominantly restricted to the gray matter seen suggests that the SEM-like phenotype has a chronic and active vascular pathogenesis that affects the watershed regions of the central branches (Figure 1) of the ventral spinal artery. The central artery supplies the majority of the spinal cord gray matter, and demonstrates variable arborization patterns in dogs, including a stem from the ventral spinal artery that bifurcates to supply both sides of the spinal cord, or segmental trunks that alternately and unilaterally perfuse the left or right sides of the gray matter (17). If the SEM-like phenotype has a vascular etiology, the bilaterally symmetric nature of gray matter necrosis would implicate that a bifurcated central artery arborization pattern is a prerequisite for its development. In a canine experimental model of compressive cervical myelopathy, reduced spinal cord blood flow was associated with progressive clinical, MRI, and neuropathological manifestations consistent with the SEM-like phenotype (13). As we did not observe any direct evidence of venous infarction, fibrocartilaginous emboli, or other causes of vasculopathy in neuropathologic examinations, we and others postulate that this may result from intermittent dynamic vascular occlusion in dogs with vertebral canal stenosis or altered vertebral column biomechanics (13).

Spinal cord compression associated with cervical disc IVDH or disc-associated CSM were associated with Cases 1–3 of canine SEM-like phenotype observed here, which is similar to humans in which extradurally compressive myelopathies, such as cervical stenotic myelopathy, are commonly observed contemporaneous pathologies in humans with SEM (9, 12, 18). Ventral slots were performed in Cases 1–3, and post-operative imaging in these dogs indicated that satisfactory decompressions were achieved using acceptably sized approach windows, which ranged between 36 and 48% of the affected vertebral widths (19, 20). However, the ventral slot approach is known to produce instability of the canine cervical spine, which could potentially contribute to dynamic vascular occlusion, reperfusion injury, or cord compression (16, 19). Dynamic spinal cord compression is a hallmark pathophysiologic phenomenon in disc-associated CSM in Dobermans, and is frequently associated with NCISC on MRI examinations (4). Repetitive concussive spinal cord injuries from dynamic disc protrusion or ligamentous hypertrophy are another possible mechanism for the development of SEM-like lesions, given the higher metabolic demands of gray matter. Serial MRI examinations performed in Case 3 were consistent with chronic, progressive SEM-like lesions associated with dynamic CSM and ligamentous hypertrophy, and both disc-associated compression and ligamentous ossification and hypertrophy were noted to contribute to vertebral canal compromise in Case 3 at necropsy. In humans, cervical stenotic myelopathy and ossification of the posterior longitudinal ligament are conditions associated with the development of SEM (9, 12).

In Case 4, which demonstrated rapidly progressive and asymmetric thoracic limb weakness and muscle atrophy from a central cord syndrome, no etiology for the SEM-like phenotype was identified. In humans, both adult-onset sporadic lower motor neuron disease (LMND) or Hirayama disease can have clinical, electrophysiologic, and SEM findings that overlap with those observed in Case 4 (21, 22). Although it is possible that the canine SEM-like phenotype with no identifiable etiology could be a manifestation of a sporadic variant of canine spinal muscular atrophy, the age of onset, clinical presentation and progression, MRI appearance, focal distribution and neuropathologic features of lesions we observed in Case 4 were not consistent with any currently described variant of canine LMND (23–25). In addition, unlike Case 4, human adult-onset sporadic LMND has a more favorable outcome with signs being very slowly progressive or even spontaneously abating in some patients (21). In Hirayama disease, forward displacement of the cervical dural sac and compressive flattening of the lower cervical cord occurs during neck flexion (22). This causes occlusion of the anterior spinal artery in the lower cervical spinal cord and subsequent ischemia in the anterior horn cells (22, 26). If left untreated, Hirayama disease will result in lower cervical spinal cord atrophy and SEM, which are considered negative prognostic indicators (26). A limitation of this case series was that neither dynamic MRI nor angiographic examinations of the cervical vertebral column were performed to assess for potential etiologies of the SEM-like phenotype.

Numerous studies in humans have attempted to associate different types and severities of ISC, including SEM, with patient prognosis. Several investigations in humans have implicated that SEM is an irreversible change associated with a poor prognosis for neurological recovery (9, 13, 26), while others suggest that SEM may be reversible depending on the cause or in the presence of early therapeutic intervention (12, 21). To further elucidate the pathogenesis of the SEM-like phenotype, performance of dynamic or vascular cervical imaging examinations should be considered if this type of ISC is identified on static MR images. We conclude that the SEM-like phenotype appears to be a manifestation of irreversible cervical spinal cord injury in dogs. Once the SEM-like phenotype involving the cervicothoracic intumescence is apparent on MRI, results of this limited case series suggests affected dogs have a poor prognosis for the preservation or recovery of thoracic limb neurological function given the longitudinally and circumferentially extensive neuronal necrosis present.

## Data Availability

All datasets generated for this study are included in the manuscript and/or the Supplementary Files.

## Ethics Statement

The study was conducted following the guidelines of Virginia Tech and the Virginia-Maryland College of Veterinary Medicine for retrospective studies with written consent obtained from participating animal owners.

## Author Contributions

JR, GK, RS, and DG participated in clinical case management. JR, GK, RS, DG, and JS reviewed neuroimaging studies. TC, DC, and JH prepared neuropathologic specimens, images, and provided neuropathological interpretations. JR and TC drafted the manuscript. All authors meet the criteria for authorship. All authors participated in the review and the editing of the manuscript.

### Conflict of Interest Statement

The authors declare that the research was conducted in the absence of any commercial or financial relationships that could be construed as a potential conflict of interest.
