# Supplementary material for: Canine Snake-Eye Myelopathy: Clinical, Magnetic Resonance Imaging, and Pathologic Findings in Four Cases
Source: Front Vet Sci. 2019 Jul 5;6:219. doi: 10.3389/fvets.2019.00219 (PMC6624786; doi:10.3389/fvets.2019.00219)
Supplement: Supplementary file 2 [file Data_Sheet_1.docx]

**Supplementary Material**

**Materials and Methods**

The medical record databases of the Virginia-Maryland College of Veterinary Medicine Veterinary Teaching Hospital and Anatomic Pathology Service were searched using the keywords canine, cervical myelopathy, cervical magnetic resonance imaging (MRI), gray matter necrosis, intramedullary signal change, intramedullary T2 hyperintensity, and (polio)myelomalacia. To estimate the prevalence of the SEM phenotype in the study population, medical records and MRI data from dogs that had a neuroanatomic diagnosis of C6-T2 myelopathy and a cervical vertebral column MRI examination performed between July of 2004 and July of 2018 were reviewed. All dogs included in this study had MRI performed using 1.5T systems (**Supplementary Table 1**), and had at the minimum the following sequences available for review: T2W transverse and sagittal, dorsal STIR, pre- and post-contrast T1W transverse and sagittal, HASTE/MYELO, and T2*GRE. Additional sequences were obtained in some cases at the discretion of the attending neurologist. In all case descriptions in this series, changes in signal intensity on MRI examinations are reported relative to the appearance to normal gray matter.

**Supplementary Table 1- MRI Protocol**

| **Parameter** | **T2W sagittal** | **T2W**  **transverse** | **T1W pre- and post-contrast sagittal** | **T1W pre- and post-contrast transverse** | **STIR**  **dorsal** | **HASTE/MYELO** | **T2*GRE**  **transverse** |
| --- | --- | --- | --- | --- | --- | --- | --- |
| **Slice thickness (mm)** | 2-3 | 3 | 3 | 3 | 3 | 3 | 3 |
| **Interslice gap (mm)** | 0.3 | 0.4 | 0.3 | 0.4 | 0.4 | 0.4 | 0.4 |
| **FOV** | 220 | 140 | 220 | 140 | 220 | 220 | 170 |
| **Matrix size** | 512x205 | 256x192 | 512x205 | 256x192 | 256x128 | 256x256 | 256x192 |
| **TR** | 3800-4200 | 3990-4440 | 500-650 | 654 | 3000 | 3800-8000 | 1020 |
| **TE** | 116-132 | 88-112 | 8-14 | 10-14 | 25-105 | 1000-1120 | 26 |
| **Averages** | 2 | 3 | 2 | 2 | 1 | 2 | 1 |
| **Other** |  |  |  |  | TI: 160 | TF: 256 | Flip angle: 20° |

**Supplemental Table 1- Key**

MRI performed on 1.5T systems (Intera, Philips, Andover, MA, USA or Signa Excite, General Electric, Chicago, IL, USA). STIR: short T1 inversion recovery sequence, HASTE/MYELO: half-Fourier acquisition single-shot turbo spin-echo, T2*GRE: gradient echo sequence. Pre: pre-contrast administration, Post: post-gadolinium contrast administration, FOV: field of view, TR: time to repetition, TE: time to echo, TF: turbo factor, TI: time to inversion

**Supplementary Video 1-** Neurological examination of Case 1, demonstrating clinical localization consistent with C6-T2 myelopathy with central cord syndrome.
